# Limited Intervention in Adult Scoliosis—A Systematic Review

**DOI:** 10.3390/jcm13041030

**Published:** 2024-02-11

**Authors:** Zuhair Jameel Mohammed, John Worley, Luke Hiatt, Sakthivel Rajan Rajaram Manoharan, Steven Theiss

**Affiliations:** Department of Orthopaedic Surgery, University of Alabama at Birmingham, Birmingham, AL 35233, USA; zjmohamm@uab.edu (Z.J.M.); jworley@uabmc.edu (J.W.); lhiatt@uabmc.edu (L.H.); srajaram@uabmc.edu (S.R.R.M.)

**Keywords:** scoliosis, degenerative, deformity, limited intervention

## Abstract

Background/Objectives: Adult scoliosis is traditionally treated with long-segment fusion, which provides strong radiographic correction and significant improvements in health-related quality of life but comes at a high morbidity cost. This systematic review seeks to examine the literature behind limited interventions in adult scoliosis patients and examine the best approaches to treatment. Methods: This is a MEDLINE- and PubMed-based literature search that ultimately included 49 articles with a total of 21,836 subjects. Results: Our search found that long-segment interventions had strong radiographic corrections but also resulted in high perioperative morbidity. Limited interventions were best suited to patients with compensated deformity, with decompression best for neurologic symptoms and fusion needed to treat neurological symptoms secondary to up-down stenosis and to provide stability across unstable segments. Decompression can consist of discectomy, laminotomy, and/or foraminotomy, all of which are shown to provide symptomatic relief of neurologic pain. Short-segment fusion has been shown to provide improvements in patient outcomes, albeit with higher rates of adjacent segment disease and concerns for correctional loss. Interbody devices can provide decompression without posterior element manipulation. Future directions include short-segment fusion in uncompensated deformity and dynamic stabilization constructs. Conclusions: Limited interventions can provide symptomatic relief to adult spine deformity patients, with indications mostly in patients with balanced deformities and neurological pain.

## 1. Introduction

Degenerative lumbar scoliosis is a common spinal pathology estimated to have a prevalence of 35.5% in patients older than 60 and an incidence of 36.7% over 12 years [1]. While not all cases are symptomatic, they can present with complaints of chronic lumbar back pain and spinal stenosis, negatively impacting the quality of life for patients who suffer from scoliosis [1]. Multiple surgical approaches are possible for the treatment and correction of adult spinal deformities, along with the alleviation of associated symptoms such as back pain or radiculopathy. These range from decompressive procedures such as laminectomies or foraminotomies up to long spinal fusion constructs. Given the morbidity associated with long-level fusions and the associated exposures and recovery, limited approaches for the treatment of spinal deformity have become an attractive option for adult spinal deformity patients. In this systematic review, we examine the different approaches and surgical techniques utilized in the treatment of spinal deformity and associated symptoms, with an emphasis on limited approaches. We examine impacts on spinal alignments and patient-related outcomes, along with patient function following operative intervention.

## 2. Materials and Methods

**Figure 1:**
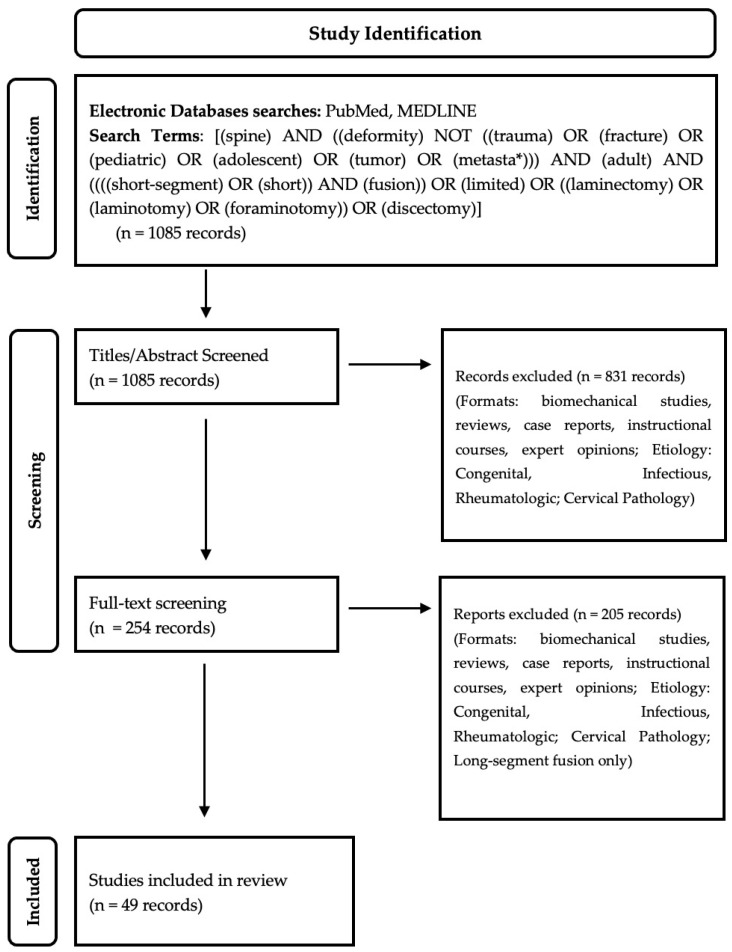
Systematic review flow diagram.

This systematic review was conducted following the Preferred Reporting Items for Systematic Reviews and Meta-Analysis (PRISMA) guidelines. This study does not have a registered protocol. We utilized PubMed and MEDLINE-indexed journals. We utilized the search terms [(spine) AND ((deformity) NOT ((trauma) OR (fracture) OR (pediatric) OR (adolescent) OR (tumor) OR (metasta*))) AND (adult) AND ((((short-segment) OR (short)) AND (fusion)) OR (limited) OR ((laminectomy) OR (laminotomy) OR (foraminotomy)) OR (discectomy))]. Our initial search yielded 1085 results. We then proceeded to perform abstract screening with assessment of each record by a single reviewer, Z.J.M., with further record confirmation with reviewer S.T. Abstract screening excluded biomechanical studies, reviews, case reports, instructional courses, and expert opinions, in addition to studies examining congenital deformities, infectious etiologies (such as tuberculosis or pyogenic spondylodiscitis), rheumatological etiologies (such as ankylosing spondylitis), and cervical pathologies or instrumented fusion. Full-text screenings were used to exclude papers focusing solely on long-construct/segment spinal fusions. The screening process is summarized in Figure 1. After abstract and full-text screening, our final analysis consisted of 49 individual studies, with 1 Level II, 37 Level III, and 11 Level IV studies (Table 1).

Data extraction was then performed by a single reviewer, Z.J.M. Data extracted for each study included study author, year of publication, study type, level of evidence, cohort size, patient population, intervention applied, and outcomes/findings. The main outcomes assessed in each study broadly included radiographic measurements (such as local and lumbar lordosis, sagittal vertebral alignment, spino-pelvic measurements including pelvic incidence, pelvic tilt, and sacral slope), symptomatic measures (such as the Visual Acuity Scale (VAS)), quality of life measures (such as the Oswestry Disability Index (ODI) or the Short Form assessments (SF-12/SF-36)), complications (such as proximal junctional kyphosis, instrument failure, and adjacent segment disease), and re-operation rates. Given the broad scope of this review and the varied patient populations covered therein, no meta-analysis of such data was able to be performed. A bias assessment of the included studies was performed using the Risk of Bias in Non-randomized Studies of Intervention (ROBINS-I) assessment tool (Supplementary Table S1).

Of note, this study focuses on limited interventions in adult spinal deformity; therefore, it becomes imperative to define what a limited intervention can look like. Cho and Kim propose a very succinct and apt definition: a limited/short intervention is one that remains confined to the deformity, not exceeding the deformity‘s upper affected vertebra or lower affected vertebra [2]. Thus, limited interventions can take multiple forms—from a single-level decompression or decompression and fusion to a multi-level decompression and fusion. While screening studies for inclusion in this analysis, we utilized an initial limit of 6 instrumented segments as an upper bound for a “short-segment” intervention and then further corroborated this with imaging to determine if the Intervention only spanned the affected vertebral levels.

## 3. Results

### 3.1. Long-Segment Fusion: Advantages and Drawbacks

Long-segment instrumented fusion, with constructs spanning the entire deformity length, has a few advantages when compared to more limited interventions. Such constructs are considered superior at radiographic correction, with multiple studies showing better post-operative radiographic outcomes in patients with long-segment fusion compared to more limited fusion. Cho et al. demonstrated that long-segment fusions (average 6.5 levels, range 4–9) had significantly greater changes in radiographic parameters, such as coronal Cobb angle, lumbar lordosis (LL), and sagittal balance (C7 plumb line), compared to more limited interventions (average 3.1 levels, range 1–5) [2]. Liu et al. found that long-segment constructs (>3 levels) resulted in significantly greater improvements in lumbar lordosis and coronal Cobb angles compared to more limited interventions (≤3 levels) [3]. Wang et al. demonstrated that more invasive, long-segment fusions (average 4.9 ± 3.1 levels) had significantly higher improvements in post-operative sagittal and coronal balance [4]. More recently, Li et al. found that full correction (average 8.1 ± 3 levels) resulted in significantly increased Cobb angle correction compared to short-segment fusion and decompression (average 2.0 ± 1.1 levels) [5]. Song et al. found in a cohort followed for 4 years that Cobb angles, lumbar lordosis, sagittal balance, and coronal balance were all significantly better following long-segment fusion (average 7.9 ± 2.1 levels) [6]. Khalifé et al. noted that long-segment constructs were significantly better at fixing Cobb angles, fractional curves, lumbar lordosis, pelvic incidence-lumbar lordosis (PI-LL) mismatch, pelvic tilt (PT), and spinosacral angles (SSA) [7]. Overall, long-segment instrumented fusion is better at restoring coronal and sagittal balance, while limited interventions remain ill-equipped to restore spinal balance.

However, while long-segment fusion is well-equipped to improve coronal and sagittal balance, it comes at the cost of high perioperative morbidity and disability. Liu et al. noted that re-operation rates were higher in their long fusion cohort compared to shorter constructs, secondary to hardware failure [3]. Cho et al. expanded on these findings, demonstrating that long-segment constructs were associated with higher rates of non-union and implant-related complications, along with higher rates of re-operation secondary to [2]. Song et al. noted significantly higher rates of complications, including nonunion, in patients undergoing long-segment instrumented fusion, findings affirmed by Khalifé et al. [6,7]. Schairer et al. demonstrated a higher risk of readmission in patients who underwent long-segment fusion [8]. While examining patients within a single deformity class, Li et al. noted significantly increased rates of complications and re-operations with long-segment constructs, with long-segment constructs suffering primarily from implant failures and proximal junctional kyphosis [5]. Hart et al. authored two studies examining the impact of instrumented levels on lumbar stiffness, as measured through the Lumbar Stiffness Disability Index (LSDI) [9,10]. In a cross-sectional study, they noted significantly lower LSDI scores in patients who underwent 1-level arthrodesis versus those who underwent 5-level arthrodesis [9]. Their follow-up study demonstrated that patients who underwent 1-level arthrodesis saw a significant decrease in LSDI, while those in the 4- and 5-level cohorts saw nonsignificant increases in LSDI [10]. Isaacs et al. noted that an increase in instrumented segments was significantly correlated with an increase in complications [11]. Conversely, Pateder et al. noted no increase in mortality with increasing fusion length [12]. Overall, long-segment fusion is associated with increased perioperative morbidity in terms of post-operative complications, readmissions, re-operations, and lumbar stiffness-related disability. Thus, limited interventions, while unable to achieve as powerful a radiographic correction, are an attractive option due the lower associated perioperative morbidity.

### 3.2. Patient Selection

While limited interventions are inadequate to provide proper sagittal and coronal alignment, their lower associated perioperative morbidity and disability lend themselves well to consideration in certain patient populations. The primary means of stratification used to determine the appropriateness of limited surgical interventions is based on a patient’s pre-operative radiographic alignment. Multiple studies have examined radiographic alignment in both limited decompressive and limited fusion procedures. In terms of decompression, Frazier et al. examined outcomes following laminectomy in patients with adult scoliosis, noting that increased pre-operative scoliosis was associated with lesser improvements in back pain upon follow-up [13]. Minamide et al. examined outcomes in patients undergoing endoscopic decompression, noting reduced symptomatic improvement in patients with a pre-operative Cobb angle of greater than 20°, along with an increasing pelvic incidence-lumbar lordosis (PI-LL) mismatch [14]. While patients still see symptomatic improvements, malalignment reduces the effects of surgery. These trends are also noted in limited fusions: Aoki et al. noted that increasing PI-LL mismatch was correlated with worse post-operative visual acuity (VAS) scores for low back pain (LBP), lower extremity pain, and lower extremity numbness [15]. Similarly, Bari et al. analyzed the impact of lordosis distribution, noting increased post-operative pelvic tilt and PI-LL mismatch, along with increased revision rates, in hypolordotic patients [16]. Bari also noted that increased pre-operative pelvic incidence was a risk factor for post-operative hypolordosis [16]. Lugue et al. noted that adult deformity patients with elevated PI-LL and sagittal vertebral axis (SVA) post-operatively had worse clinical outcomes, with lower Japanese Orthopaedic Association (JOA) and Short Form 36 (SF-36) scores [17]. Overall, patients with uncompensated or unbalanced deformities tend to not see as powerful symptomatic improvements as those with balanced deformities following limited interventions. Thus, such interventions are more appropriate for patients with balanced deformities.

In this review, we discuss two broad categories of interventions: decompressive procedures, including discectomy, laminectomy, and foraminotomy; and fusion procedures, including interbody fusions. These two procedures are best equipped for the treatment of differing deformity symptoms. Liang et al. mention the classification of patients based on their symptomatic complaints into two categories: neurogenic pain, resulting from central canal, lateral recess, and foraminal stenosis; and axial pain, resulting from muscle fatigue secondary to sagittal imbalances [4]. Treatment strategies for these two distinct problems differ. Patients with neurogenic symptoms often present with radicular or cauda equina-like pain secondary to the central canal, lateral recess, and foraminal stenosis [3,4]. In such patients, decompressive procedures can be considered, with simple nerve root decompression reserved for purely radicular pain and posterior element manipulation reserved for cases with segmental canal stenosis [3]. Patients with axial pain present with low back pain secondary to muscle pain. Such symptoms can be divided into two separate types: primary imbalance due to malalignment and secondary imbalance due to stenosis, which leads to paraspinal muscular fatigue and loss of lumbar lordosis [4]. In patients with a primary, uncompensated deformity, long fusion is necessary for treatment of the deformity and restoration of spina alignment, which will result in the resolution of symptoms [3,4]. Wang et al. discuss treatment in patients with compensated deformities—such patients often suffer more from neurogenic symptoms than axial-based symptoms, and axial symptoms are often secondary to neurological deformity; thus, such patients can also undergo either decompression or fusion for symptomatic treatment, depending on the symptoms seen [4]. One other consideration to take note of is the presence of cephalad-caudad directional stenosis, colloquially referred to as “up-down stenosis” [30]. Such patients require fusion with interbody support for adequate decompression [30]. Based on the aforementioned considerations, we can begin to develop a treatment algorithm for spinal deformity—simple neurological complaints with radicular pain are more appropriately treated with decompressive procedures. In cases of compensated deformity and/or cephalad-caudad stenosis, then fusion becomes necessary for symptomatic treatment.

### 3.3. Decompression

Decompressive procedures are among the least-invasive procedures available for the surgical treatment of spinal deformities. Discectomy provides a minimally invasive technique to treat neurogenic symptoms secondary to disc herniation. Pugely et al. found that discectomy carries the lowest re-admission rates in spine surgery, in stark contrast with deformity correction [18]. Thus, discectomy presents an attractive option for limited intervention in adult deformities. The overwhelming majority of papers found in this analysis examined one specific approach to discectomy—percutaneous transforaminal endoscopic discectomy. Kapetanakis et al. demonstrated that this approach leads to significant improvements in patient-reported outcomes (PROs) such as the visual analog scale (VAS) and Oswestry Disability Index (ODI), with patients experiencing improved quality of life following discectomy [19]. Some studies have further expanded upon surgical techniques noted, devising novel approaches to discectomies, especially at the lumbosacral junction. Kim et al. proposed an interlaminar contralateral endoscopic discectomy with overall similar outcomes to transforaminal approaches, with lower rates of post-operative dysesthesia noted in the inter-laminar approach [20]. Meanwhile, Bai et al. demonstrated a trans-iliac approach for approaching such pathology, with similar outcomes noted to open approaches [21]. Telfeian et al. examined the applicability of discectomy in adult deformity in patients previously treated for lateral vertebral subluxation, a common finding in adult degenerative scoliosis, showing clinically significant improvements in both ODI and VAS scores [22]. Methods such as transforaminal endoscopic discectomy benefit from minimal disruption to the spinal posterior elements, utilizing a minimally invasive foraminotomy to access the affected disc, leaving the ligaments and musculature of the spine mostly intact [19,22]. While more studies are needed on the topic, discectomy is appropriate in patients with deformities who suffer from radicular symptoms due to herniated discs, and such treatment can provide symptomatic relief and clinical improvement in affected patients.

Discectomies are only a part of the spine surgeon’s toolkits; surgeons can also perform more involved surgical procedures such as laminectomies and foraminotomies, with manipulation of the posterior spinal anatomy for further decompression of the spinal canal. Overall, such procedures are associated with symptomatic improvement, with a study by Madhavan et al. showing significant improvements in patient-reported outcomes, such as VAS, following foraminotomy [23]. Brodke et al. noted that compared to less invasive modalities, such as interspinous spacers, laminectomies show better improvements in VAS, lower post-operative mortality, and recurrence, along with similar improvements in VAS for laminectomy and fusion patients [24]. Minamide et al. noted that patients undergoing endoscopic decompression had overall significant improvements in clinical outcomes, such as JOA scores [14]. Hasan et al. further expanded on the role of minimally invasive or endoscopic interventions in decompression, finding similar outcomes between both endoscopic and MIS interventions, albeit with lower complication rates in endoscopic interventions [25]. Decompressive procedures can provide symptomatic relief and clinical improvement in patients suffering from neurogenic pain, relieving pressure on neural structures. However, in some cases, especially secondary to deformity resulting in posterior element impingement of neural structures, realignment becomes necessary to fully decompress the spine.

### 3.4. Fusion

As previously mentioned, decompression is only appropriate for neurogenic symptoms resulting in cauda equina- or radicular-pattern pain [14,25]. However, axial pain resulting from mechanical instability will remain relatively unchanged as a result of decompressive procedures [14]. In such cases, spinal fusion, in addition to decompression, may become necessary. While decompressive procedures are well-equipped to provide some decompression of posterior neural elements, in certain cases, realignment becomes necessary for full neural decompression. In addition, in axial pain, which results from muscular fatigue from sagittal imbalances, fusion can provide some level of sagittal correction [4]. In cases of compensated deformity, where limited fusion constructs are most appropriate, fusion can stabilize decompression levels and prevent further deformity occurrence [3,4]. In comparison to purely decompressive interventions, decompression and fusion surgeries have a larger body of literature regarding their use. Fusion constructs often consist of interbody fusion and/or posterior spinal instrumentation, allowing for both the restoration of spinal radiographic parameters and rigid fixation of the deformity. This review focuses on limited interventions and thus will focus on short-segment fusions, which have been defined as spanning only the affected vertebral segments, with the upper instrumented vertebra and lower instrumented vertebra falling within or at the ends of the deformity [2]. Such segments need not span only the deformity—rather, they can also span symptomatic levels, with levels responsible for neurogenic or stenotic symptoms undergoing decompression and fusion as well.

#### 3.4.1. Short-Segment Fusion: Does It Provide Relief?

As previously noted, long-segment fusion has been shown to have a stronger ability to provide both sagittal and coronal correction of spinal deformity compared to short-segment fusion and decompression constructs [2,3,4,5,6,7]. However, long-segment instrumented fusion has also been shown to carry a significant burden of post-operative morbidity and disability in comparison to more limited interventions [2,3,5,6,7,8,9,10,11]. Thus, in cases of compensated deformity, where given a balanced deformity, sagittal correction is not the primary aim of treatment, short-segment fusion and decompression can provide adequate symptomatic relief [4]. When juxtaposed with long-segment constructs in the setting of balanced deformities, short-segment constructs have been shown to provide similar clinical outcomes, both with regards to symptomatic improvement and functional outcomes. Numerous studies demonstrate similar functional and symptomatic outcomes between short-segment and long-segment instrumented fusion. Song et al. noted no significant differences in VAS-Back and VAS-Leg scores between a long-segment and short-segment cohort [6]. Khalifé et al. echoed these results: while they found a significantly lower VAS score for radicular pain with long fusion, they noted similar VAS-Back, ODI, and Scoliosis Research Society (SRS)-30 scores [7]. Liu et al. also noted similar outcomes in ODI following long-segment and short-segment fusions, albeit with greater improvement in ODI with long-segment fusions [3]. Wang et al. showed similar improvements in ODI and SRS-22 scores, albeit between a compensated cohort receiving short-segment fusion and a decompensated cohort receiving long-segment fusion [4]. Uribe et al. showed that despite having short construct lengths, MIS techniques could result in similar clinical and radiographic outcomes to open surgery with lesser re-operation rates, blood loss, and hospital stay [26]. Deukmedjian et al. noted that in patients with compensated deformity, utilizing less invasive means and constructs led to significant improvements in ODI and VAS scores [27]. Cho et al. demonstrated that short- and long-segment fusion achieve similar changes in ODI post-operatively [2]. Park et al. demonstrated that short-segment fusion in patients with coronal Cobb angles of 40° or less leads to significant improvements in ODI, VAS, and SF-36 scores [28]. Nakajima et al. noted significant improvements in ODI, JOA score, and Numerical Rating (NRS) score following short-segment fusion [1]. Given the aforementioned body of evidence, short-segment instrumented fusion, while underpowered to provide radiographic realignment, shows equivalent and acceptable patient-related outcomes to long-segment fusion in patients with compensated deformities in adult scoliosis.

#### 3.4.2. Role in Uncompensated Spinal Deformity?

We previously mentioned the concepts of spinal balance and its role in patient selection for limited intervention. The traditional literature has demonstrated that short-segment fusion. may not demonstrate adequate patient outcomes following intervention. Nakajima et al. found that while short-segment surgery saw overall improvements in PROs and radiographic outcomes, measurements such as PI-LL mismatch remained high, and patients who required re-operation often had pre-operative uncompensated deformities such as lumbar kyphosis [1]. Deukmedjian et al. noted that surgical undertreatment for larger deformities can lead to worsening sagittal balance [27]. Given the risks of progression and future re-operation, the traditional viewpoint has remained. Recently, some studies have examined outcomes in patients with decompensated deformities who underwent short-segment instrumented fusion. Liang et al. examined outcomes in deformity patients with limited sagittal alignment correction versus full correction and concluded that despite worse sagittal alignment in the limited correction group, clinical outcomes such as ODI and JOA scores did not differ significantly [29]. However, the literature supporting this view is novel and thus limited, so no conclusions can be drawn regarding short-segment constructs in uncompensated deformity. Thus, short-segment instrumented fusion in adult spinal deformity patients is currently most appropriate for non-neurogenic pain in a compensated/balanced spine, although future studies may clarify its use in uncompensated deformities.

#### 3.4.3. Fractional Curve Treatment: A Means of Foraminal Decompression

Some specific approaches and variations to short-segment instrumented fusion exist in the literature. Traditional treatment of scoliosis focuses on correction of the primary curve, with instrumentation spanning the apex of the primary curve. One treatment approach surgeons can utilize is the correction of the fractional curve, which represents the secondary scoliotic curve at the lumbosacral junction and can be a source of neurogenic pain secondary to loss of foraminal height [29]. Amara et al. compared outcomes in adult scoliosis patients between fractional curve correction versus fusion to the lower or upper thoracic spine [30]. They found that while longer fusion constructs were better at providing radiographic correction, fractional curve treatment led to overall lower blood loss, length of hospital stay, medical complications, and non-extension revision operations [30]. A study by Chou et al. examined differences in approaches utilized in fractional curve correction, comparing an open approach versus a minimally invasive (MIS) approach [31]. Their findings indicated that overall, MIS approaches resulted in lower levels of blood loss and greater improvements in VAS Leg scores, which are impacted by neurogenic pain, despite fewer patients undergoing nerve root decompression [31]. Fractional curve treatment can provide symptomatic relief while reducing perioperative morbidity in comparison to traditional techniques.

#### 3.4.4. Short-Segment Fusion: Potential Pitfalls?

While short-segment fusion has been shown to provide similar clinical outcomes to long-segment fusion with reduced perioperative morbidity, one of the most concerning complications of shorter constructs remains adjacent segment disease, with increased degeneration seen in the remaining curve [2]. Both Liu and Cho demonstrated that short-segment fusion had increased rates of adjacent segment disease, albeit not attaching any significant statistics to these findings [2,3]. Song et al. noted an increased rate of adjacent segment disease in short-fusion constructs, although this did not reach significance [6]. Interestingly, Khalifé et al. noted a higher rate of adjacent segment disease in the long fusion cohort, although no significance was able to be determined [7]. One item to note is the difference between radiographic and clinical adjacent segment disease. Song et al. noted that out of 14 patients with adjacent segment disease, only 4 (28.57%) had clinical symptoms [6]. Cho et al. noted only adjacent segment disease patients with clinical symptoms, noting only proximal disease in short fusions [2]. Liu et al. defined adjacent segment disease based on radiographic findings but noted that patients with radiographic findings had significant clinical complaints [3]. Overall, the evidence presented in this review remains mixed—while most studies show a higher incidence of adjacent segment disease, there are not much data on the significance of these findings and on whether radiographic disease leads to clinical findings. More data are needed to provide clarity on the matter.

One concern with short-segment fusion concerns the progression of deformity following fusion. Amongst the previously stated studies, those by Song, Liu, Khalifé, and Nakajima all contained data regarding differences in radiographic outcomes upon extended follow-up [1,3,6,7]. Liu et al. noted that patients undergoing short-segment fusion demonstrated a significant loss in lumbar lordosis at final follow-up compared to pre-operatively and some progression in Cobb angle as well, although there were approximately 6 and 5 years between the time points measured, respectively [3]. Nakajima et al. noted some correctional loss at follow-up but noted that the median loss was extremely small, in the single digits [1]. Song et al. noted some loss of radiographic correction in their cohort, while the long-segment fusion cohort better maintained correction over 5 years [6]. Most of the studies quoted show some progression of disease and loss of correction with time, thus raising concerns for future re-operations. However, Nakajima et al. note that the amount of Cobb angle progression seen after short-segment fusion is similar to natural progression, arguing that short-segment fusion does not lead to accelerated degeneration [1]. Moreover, Song et al.’s data demonstrated that correctional loss still resulted in better radiographic alignment in terms of Cobb angles and lumbar lordosis in short-segment fusion, albeit with loss of coronal and sagittal balance [6]. Overall, while studies do show loss of correction with short-segment fusion over time, the absolute loss over time remains in the single digits and often corresponds to the natural progression of the disease.

Given the potential for correction loss, one question that arises is the impact of short-segment constructs on future revisions. A study by Kasliwal et al. examined outcomes in patients with adult scoliosis following deformity correction, comparing patients undergoing re-operation with previous short-segment instrumented fusion with patients undergoing their first spinal procedure [32]. While patients with a previous operation had a near-significantly higher number of instrumented levels and higher blood loss, overall there were no significant differences between the two cohorts [32]. Both cohorts had similar outcomes in terms of both radiographic and clinical outcomes between the two cohorts. In addition, there was no significant difference in the post-operative complication rates between the re-operative and control cohorts [32]. Thus, while there are some differences in surgical parameters, re-operative patients have similar outcomes to first-time operative patients.

#### 3.4.5. Interbody vs. Posterior Instrumentation Only

Historically, pedicle screw instrumentation was considered effective in the treatment of adult deformities—Zurbriggen et al. demonstrated that posterior instrumentation in adult scoliotic deformities results in mostly good and excellent post-operative results, with correction of scoliotic Cobb angle and augmentation of lumbar lordosis [33]. However, more recent literature has shown interbody constructions, which augment pedicle screw constructs with interbody spaces, to have equivalent outcomes in deformity correction. Feng et al. examined outcomes in patients with isthmic spondylolisthesis undergoing posterior instrumentation versus posterior lumbar interbody fusion [34]. They noted similar radiographic correction and clinical outcomes in both cohorts, albeit with a lower incidence of pseudoarthrosis in the interbody cohort [34]. Other studies have examined the independent impacts of interbody-based constructs. Johnson et al. noted post-operative improvements in VAS, ODI, and SF-36, along with a significant increase in segmental lordosis and a significant decrease in coronal Cobb angles in patients with degenerative disc disease and degenerative scoliosis [35]. Anand et al. examined MIS approaches in interbody instrumented fusion and noted significant improvements in coronal Cobb angles, VAS scores, and Treatment Intensity (TIS) scores post-operatively [36]. Hasegawa and Homma showed that posterior lumbar interbody fusion could be used for sagittal and coronal deformity correction, with improvements in clinical outcomes based on JOA scores [37]. Dakwar et al. showed improvements in ODI and VAS with lateral lumbar interbody fusion for adult scoliosis patients [38]. In summary, interbody fusion is an effective means of providing symptomatic relief to patients with adult scoliotic deformities and should be utilized as appropriate.

In terms of interbody fusion, multiple approaches exist, including anterior (ALIF), lateral (LLIF), transforaminal (TLIF), and posterior (PLIF). Given the multitude of [39] approaches and constructs possible, surgeons have to consider the benefits and drawbacks of each interbody spacer type for their patients. A study by Ahlquist et al. examined radiographic measurements in patients undergoing single-level lumbar interbody fusion, and they demonstrated that ALIF and TLIF techniques were superior at restoration of segmental lordosis, lumbar lordosis, disc heights, and foraminal heights, with TLIF also able to aid in restoration of these parameters to a lesser extent [40]. Lee et al. examined the usage of different surgical approaches while performing ALIF in adult scoliosis patients and also noted a significant number of patients with post-operative restoration of normal PI-LL, along with improvements in lumbar lordosis, but they also noted that ALIF can potentially present with greater approach-related morbidity [39]. Given that LLIF and ALIF have been shown to provide better restoration of spinal alignment, they may be more appropriate interventions for the treatment of adult scoliosis. Of note, LLIF procedures cannot be performed at the L5-S1 junction given anatomical constraints, and thus cannot be used for treatment of the fractional curve at that specific level.

One technical note with regards to surgical approaches involves the use of minimally invasive techniques versus open approaches. The previously mentioned study by Lee et al. noted that open approaches, including those that utilize osteotomies, resulted in greater sagittal correction over a percutaneous approach, thus indicating that open approaches may be better equipped for sagittal restoration [39]. However, they also noted reduced intraoperative blood loss and blood transfusion in the percutaneous approach, along with similar improvements in ODI and VAS scores compared to other approaches [39]. Other papers have highlighted similar benefits of minimally invasive approaches. Anand et al. noted reduced intra-operative blood loss and morbidity in patients undergoing MIS LLIF, along with significant improvements in Treatment Intensity Score (TIS), VAS, ODI, SF-36 scores, and Cobb angle post-operatively [41]. Lo et al. examined mini-open and MIS TLIF approaches in single-level fusion for adult degenerative disorders and found improved post-operative VAS scores, reduced blood loss, and shorter hospital stays in the mini-open and MIS cohorts [42]. Seng et al. noted lesser blood loss, morphine usage, and shorter hospital stays in their MIS cohort, along with earlier ambulation and similar post-operative outcomes to open approaches [43]. Isaacs et al. noted that LLIF procedures that were entirely MIS had lower complication rates than procedures involving open posterior approaches [11]. Given the lower morbidity surrounding MIS surgeries, which have also shown equivalent outcomes to open approaches, MIS approaches may present as a more appropriate approach in limited deformity correction.

#### 3.4.6. Need for Decompression?

While the vast majority of constructs discussed thus far have utilized a combination of fusion and decompression, recent literature has raised an interesting question regarding the utility of decompression in the setting of interbody fusion. Given that interbody fusions can restore disc heights and foraminal heights, there is a possibility that interbody fusion alone is sufficient for the resolution of neurogenic symptoms. There are a few studies that have examined this hypothesis specifically. Alimi et al. examined unilateral stenosis in adult deformity patients and proposed treating it with a unilateral LLIF without decompression, noting significant increases post-operatively in both stenotic and contralateral formaminal and disc heights sustained on follow-up [44]. Similarly, they noted significant improvements in VAS-leg pain and buttock, which correlate with neurogenic symptoms, along with VAS-Back and ODI scores [44]. Tani et al. examined the impact of using anterior column realignment (ACR) in conjunction with LLIF and percutaneous pedicle screw instrumentation on neural anatomical elements, showing significant improvements in sagittal alignment, disc height, foraminal height, dural sac cross-sectional area, and ODI, along with decreased ligamentum flavum thickness and disc bulge thickness [45]. As previously mentioned, Chou et al. looked at the treatment of the fractional curve with interbody spacers and found similar outcomes to posterior open approaches, including improvements in VAS-Leg pain without surgical decompression [31]. Thus, interbody fusion can provide neurological decompression through the restoration of disc height and foraminal height.

While interbody spacers may restore disc and foraminal height and reduce neurological compression, what advantages do such procedures have over fusion and decompression combinations? Multiple studies have indicated that surgical decompression, when combined with fusion, can lead to increased post-operative complications. Elsamadicy et al., in a retrospective cohort of 874 spinal deformity patients, found that fusion procedures that incorporated laminectomies were associated with significantly higher rates of intra-operative blood loss, blood transfusions, and durotomies, along with increased intensive care unit admissions and rates of altered mental status, urinary tract infections, wound drainage, and instrumentation failure [46]. In another study design, Brodke et al. examined outcomes between laminectomies and fusions with laminectomies and found that the fusion cohort had significantly higher rates of early and late adjacent segment disease and significantly lower VAS-Leg and patient satisfaction [24]. Of note, avoiding decompressive procedures maintains posterior spinal anatomy by avoiding manipulation of the facet joints and posterior ligamentous structures, leading to improved overall spinal stability in patients post-operatively. Posterior element manipulation can lead to higher complication and revision rates in the setting of interbody fusion, thus providing some support to fusion-only constructs.

### 3.5. Dynamic Stabilization—A Potential Future Option?

While the aforementioned categories cover the vast majority of limited interventional treatments for adult spinal deformity, our literature search uncovered a few more techniques that did not quite fall into the aforementioned categories. Such approaches mostly utilize dynamic fixation and stabilization. Traditional instrumented fusion allows for the correction of sagittal and coronal deformities, helping restore spinopelvic parameters, but it also alters spine biomechanics, leading to an increased incidence of adjacent segment disease [47]. Dynamic fixation, utilizing ligament-and-screw constructs, can help preserve normal spinal biomechanics. Kanayama examined the use of Graf ligamentoplasty, consisting of pedicle screws and looped, braided polyester bands, to provide dynamic stability and found that Graf ligamentoplasty maintained segmental motion but was associated with poor clinical outcomes [47]. They concluded that Graf ligamentoplasty was inappropriate for the treatment of adult degenerative scoliosis and laterolisthesis [47]. Subsequently, Di Silvestre et al. examined the usage of the Dynesys system in adult degenerative scoliosis, reporting statistically significant improvements in ODI, RMDQ, and VAS scores for leg pain and back pain, along with statistically significant corrections in scoliosis Cobb angle and anterior vertebral translation [48]. Zhao et al. examined short-segment instrumented fusion with proximal dynamic stabilization with the Wallis system, an interspinous spacer and a fixator and noted improvements in ODI and VAS scores along with no adjacent segment disease cephalad to fusion, but also noted limited radiographic correction [49]. Given the relatively small body of literature, with limitations in statistical power, regarding the usage of dynamic fixation in adult deformity, no concrete conclusions can be drawn regarding its efficacy. However, dynamic fixation remains a potential intervention for adult deformity, allowing for radiographic correction while maintaining segmental motion.

## 4. Discussion

This review delves into the different treatment options available for limited correction of adult spinal deformities. This field is ever-evolving, with new studies published yearly, further evolving our understanding of the field. Traditional deformity treatment relied heavily on long-segment constructs, which provided appropriate radiographic correction but were associated with significantly increased levels of post-operative morbidity and lumbar stiffness. Given these drawbacks, more limited interventions, when appropriate, can provide similar symptomatic and functional recovery without the associated morbidity of long-segment fusion. Such approaches are most appropriate in patients with compensated or balanced deformities, as limited interventions cannot provide significant improvements in radiographic parameters following surgical intervention. Thus, spinopelvic alignment, through the use of PI-LL, along with coronal deformity and sagittal deformity, should be considered when evaluating patient eligibility for limited interventions.

Given a compensated/balanced deformity, the next qualifier for intervention is based on the quality of pain and symptoms experienced by the patient. We previously categorized scoliotic pain into two categories—neurogenic and mechanical. Neurogenic pain occurs because of neurological compression, presenting with cauda equina- or radicular-like pain and deficits. In such patients, given the neurogenic etiology of their pain, decompression via discectomy or manipulation of the posterior spinal elements can provide symptomatic relief and is an appropriate first option for treatment. In patients with compensated deformity and low back pain, the underlying etiology of their pain relates muscular pain secondary to neural impingement. As such, short-segment fusion, which can provide a more powerful decompression, is a more appropriate option for patient treatment. Similarly, for patients with cephalad-caudad stenosis, decompression may not be enough to resolve symptoms, and fusion with interbody support will be necessary to provide relief.

While limited interventions may lack the ability of long-segment fusions to provide lasting sagittal re-alignment, they present immediate benefits in terms of perioperative morbidity, along with lesser stiffness-related disability following surgery. While radiographic outcomes in limited interventions are often significantly lesser than in long-segment interventions, functional and symptomatic outcomes—measured by patient-related outcomes such as ODI, VAS, JOA score, or SF-36—often show no significant differences in limited interventions when compared to long-segment fusions. Thus, they remain an appropriate option for compensated deformities. That being said, more recent literature argues for the efficacy of short-segment constructs in uncompensated deformities, noting that similar clinical outcomes are seen in such patients. However, more studies are needed to better understand the usage of short-segment instrumented fusion in uncompensated deformity. One specific short-segment construct looks at the treatment of the fractional curve, which presents at the lumbosacral junction—such constructs are well-suited to reduce pressure on neurological structures and improve neurogenic pain. Short-segment fusion also does not preclude future extension or operations—such constructs do not lead to increased morbidity in re-operative patients compared to treatment-naive patients.

Many papers today describe the usage of interbody constructs, which can provide improvements in patient outcomes while also reducing morbidity and operative complications due to more minimally invasive exposures. Interbody spacers lend themselves well to MIS constructs, which may not provide the same sagittal correction as open constructs but provide clinical improvements and reduce perioperative morbidity. In addition, interbody spacers help restore both disc and foraminal height, thus reducing pressure on the spinal cord and exiting nerves. Interbody spacers alone can provide adequate decompression without posterior element manipulation, which has been shown to increase complication rates compared to constructs that do not utilize decompression.

One of the more novel constructs being studied is dynamic fixation and stabilization, which use artificial bands and posterior instrumentation to provide stabilization of the spine while preserving movement. Some earlier constructs proved ineffective in scoliotic patients, but later constructs have shown promise both as a primary treatment and as an adjuvant to fusion to reduce adjacent segment disease incidence. However, there is a dearth of literature on this matter compared to other topics discussed, and this provides a future avenue for further work on the correction of scoliotic deformity.

### Limitations

Our study is inherently limited by the evidence used for it. Given that the vast majority of the evidence we have compiled has a Level III level of evidence and a ROBINS-I score of moderate, most of the evidence we provide can help with drawing conclusions regarding treatment plans but does not consist of many randomized control trials, the golden standard of evidence. Our review process was also limited to MEDLINE-indexed journals, thus missing those indexed on SCOPUS, Web of Science, Google Scholar, and other indexing engines. Nonetheless, our paper provides a broad overview of considerations and practices for limited spine intervention in adult deformity patients.

## 5. Conclusions

Traditional adult scoliosis treatment has relied heavily on long-segment instrumented fusion, which provides sagittal realignment but comes at the cost of significant perioperative morbidity and spinal stiffness. In this review, we highlight the different limited treatment options available for scoliosis patients. Decompressive procedures such as discectomy or laminotomy/foraminotomy are best reserved for patients with neurogenic pain, while short-segment fusion is better suited for patients with neurologic symptoms secondary to compensated deformity, decompression of neural elements, stabilization of a short spinal segment, and relief of up-down stenosis. Such patients often see significant clinical improvement after surgical intervention. Interbody spacers can help provide restoration of normal spinal alignment and can potentially aid in the decompression of affected levels. Patients with an uncompensated deformity, as well as those with significant deformity-related symptoms and pain despite compensation, may be candidates for long-segment constructs. Future studies can examine short-segment fusion in uncompensated deformity and the potential for dynamic stabilization in adult scoliosis.

## Figures and Tables

**Table 1:** List of included studies.

Author, Year	Study Design	*n*	Level of Evidence	Cohort	Intervention	Findings
Nakajima et al., 2022 [1]	Retrospective Cohort	26	III	Patients with adult spinal deformity	Three-level, Two-stage limited lumbar fusion	Significant improvements in coronal Cobb angle, C7 SVA, and PI-LL mismatch
Cho et al., 2008 [2]	Retrospective Cohort	50	III	Patients with degenerative lumbar scoliosis	Short fusion VERSUS Long fusion	Significantly better coronal Cobb correction, coronal imbalance, and lateral listhesis correction in long fusion cohort; higher rates of early complications in long fusion, adjacent segment disease in short fusion; no significant difference in post-operative ODI
Liu et al., 2009 [3]	Retrospective Cohort	112	III	Patients with degenerative lumbar scoliosis	Simple nerve decompression VERSUS short fusion and decompression VERSUS long fusion and decompression	Significantly greater improvement in lumbar scoliosis and lordosis in long fusion cohort over short fusion and simple decompression cohorts; significantly greater improvement in ODI in the long fusion cohort compared to the short fusion and simple decompression cohorts; increased rates of ASD in short fusion cohort—only 53.8% of patients symptomatic
Wang et al., 2016 [4]	Retrospective Cohort	108	III	Patients with degenerative lumbar scoliosis with associated lumbar stenosis	Simple nerve decompression VERSUS short fusion and decompression VERSUS long fusion and decompression	Significant differences between cohorts in post-operative coronal C7 plumb line, sagittal C7 plumb line, and rotational olisthesis; significant difference in post-operative final ODI between cohorts
Li et al., 2021 [5]	Retrospective Cohort	136	III	Patients with adult spinal deformity	Focal decompression VERSUS short-segment fusion VERSUS full scoliosis correction	Decompression and short fusion with significantly shorter surgical duration, less blood loss, shorter hospital stay; amongst MISDEF2 Class II patients, patients undergoing full correction had significantly higher rates of perioperative complications and revision surgery
Song et al., 2022 [6]	Retrospective Cohort	78	III	Patients with adult degenerative scoliosis	Short-segment limited fixation VERSUS long-segment radical fixation	No significant differences between cohorts in long-term complications and re-operations; long-segment group had significantly better coronal Cobb, lumbar lordosis, and sagittal balance; long-segment group had significantly higher implant-related complications
Khalifé et al., 2023 [7]	Prospective Cohort	154	IV	Patients with scoliosis and lumbar stenosis	Lumbar decompression VERSUS short fusion and decompression VERSUS long fusion with deformity correction	Long fusion cohort with significant improvement in ODI, VAS, SF-12, SRS-30 scores at 2 years; significant increases noted in fractional curve Cobb in short fusion and C7 coronal tilt in decompression cohorts; long fusion had highest overall complication rates and revision rates
Schairer et al., 2013 [8]	Retrospective Cohort	836	III	Patients with adult spinal deformity	Spine fusion	Higher rates of readmission in patients with long fusion; risk factors for readmission were longer fusion length, higher illness severity, and medical comorbidities
Hart et al., 2013 [9]	Cross-Sectional	93	III	Patients who previously underwent lumbar spine fusion	Lumbar spine fusion	LSDI scores significantly different between 1-level and 5-level arthrodesis group; LSDI and ODI significantly correlated
Hart et al., 2014 [10]	Prospective Cohort	62	II	Patients with lumbar degenerative disease or spinal deformity	Lumbar spine fusion	All cohorts saw significant decreases in ODI following surgery; patients undergoing 1-level and 5+-level surgery saw significant improvements in physical composite score; patients with 1-level fusion saw significant decrease in LSDI; 3, 4, and 5+-level saw nonsignificant increase in LSDI
Isaacs et al., 2010 [11]	Prospective Cohort	107	IV	Patients with degenerative scoliosis	Extreme lateral interbody fusion ± posterior fixation	12.1% major complication rate, which compares favorably to previous literature
Pateder et al., 2008 [12]	Retrospective Cohort	361	III	Patients with spinal deformity	Deformity correction	Strong association between ASA score and mortality; no association between levels of fusion and mortality
Frazier et al., 1997 [13]	Prospective Cohort	90	IV	Patients with spinal deformity and spinal stenosis	Laminectomy	Pre-operative scoliosis is associated with decreased improvement in back pain following laminectomy
Minamide et al., 2017 [14]	Prospective Cohort	122	III	Patients with degenerative lumbar scoliosis with associated lumbar stenosis	Microendoscopic laminectomy or foraminotomy	Significant improvement in VAS-low back pain; clinical outcomes in foraminal stenosis related to pre-op Cobb angle and scoliosis progression
Aoki et al., 2015 [15]	Retrospective Cohort	52	III	Patients with degenerative lumbar disease	1 or 2 level TLIF	Significant correlation between post-operative PI-LL mismatch and VAS scores for low back pain, lower extremity pain, and numbness
Bari et al., 2021 [16]	Retrospective Cohort	149	III	Patients with degenerative lumbar disease	Fusion surgery ≤ 4 levels	Hypolordotic group had increased odds of 1-year revision surgery; linear correlation between pre-operative pelvic incidence and post-operative lordosis distribution
Lugue et al., 2020 [17]	Retrospective Case-Control	119	III	Patients with degenerative lumbar disease	L4/L5 PLIF	Significant increase in local lordosis, correlated with increase in lumbar lordosis; high PI-LL and SVA cohorts had decreased clinical outcomes
Pugely et al., 2014 [18]	Prospective Cohort	15,668	III	Patients undergoing lumbar spine surgery	Lumbar spine surgery	Lowest risk of readmission with discectomy and highest risk with deformity surgery
Kapetanakis et al., 2017 [19]	Prospective Cohort	76	III	Patients with lumbar disc herniation	Percutaneous transforaminal endoscopic discectomy	Significant improvements seen in all domains of SF-36 scores
Kim et al., 2021 [20]	Retrospective Cohort	100	IV	Patients with lumbar disc herniation	transforaminal endoscopic lumbar foraminotomy and discectomy VERSUS interlaminar contralateral endoscopic lumbar foraminotomy and discectomy	Interlaminar approach associated with reduced rates of post-operative dysesthesia; both cohorts had favorable clinical outcomes
Bai et al., 2017 [21]	Prospective Cohort	39	III	Patients with lumbar disc herniation	Inter-vertebral approach VERSUS trans-iliac approach	No significant differences in operative time and post-operative VAS scores between the cohorts
Telfeian et al., 2018 [22]	Case Series	4	IV	Patients with lumbar disc herniation in setting of lateral lumbar listhesis	Percutaneous transforaminal endoscopic discectomy	Most patients saw improvements in ODI and VAS sustained for 1 year follow-up
Madhavan et al., 2016 [23]	Retrospective Cohort	16	III	Patients with scoliotic deformity and unilateral radicular pain secondary to foraminal stenosis	Endoscopic foraminal decompression surgery	Significant improvement in VAS for radicular leg pain post-operatively
Brodke et al., 2013 [24]	Retrospective Cohort	90	III	Patients with lumbar stenosis in setting of spinal deformity	Interspinous spacer VERSUS laminectomy only VERSUS laminectomy and short-segment fusion	Significantly higher recurrence rate in interspinous spacer cohort; laminectomy alone cohort had highest 5-year survival on Kaplan-Meier analysis
Hasan et al., 2019 [25]	Prospective Cohort	45	III	Patients with degenerative spinal deformity with associated lumbar stenosis	Full-endoscopic VERSUS minimally invasive unilateral laminotomy for bilateral decompression	Endoscopic cohort had significantly shorter hospital stay, lower adverse events, and improved early ODI scores
Uribe et al., 2017 [26]	Retrospective Cohort	84	III	Patients undergoing adult spinal deformity correction	Minimally invasive VERSUS open approaches	MIS cohort had shorter construct lengths, lower blood loss, and shorter hospital length of stay
Deukmedjian et al., 2013 [27]	Retrospective Cohort	27	III	Patients who underwent surgical correction of adult degenerative scoliosis	Lumbar interbody fusion with augmentation dependent on deformity severity	Most cohorts showed improvements in radiographic and clinical outcomes; patients who were undertreated did not show significant improvements
Park et al., 2013 [28]	Retrospective Cohort	105	IV	Patients with adult lumbar degenerative scoliosis with a coronal Cobb angle of <40°	Decompression and instrumented fusion	Significant improvements noted in ODI, SF-36, and VAS scores post-operatively
Liang et al., 2020 [29]	Retrospective Cohort	58	III	Patients with adult degenerative scoliosis	Deformity correction surgery	Patients with limited correction in setting of sagittal imbalance had significantly worse radiographic outcomes but demonstrated no significant differences in coronal Cobb angles, ODI, or VAS
Amara et al., 2019 [30]	Retrospective Cohort	99	III	Patients with adult scoliosis	Fractional curve limited fusion VERSUS instrumentation to lower thoracic spine VERSUS instrumentation to upper thoracic spine	Fractional curve treatment with significantly lower rates of medical complications, lower blood loss, shorter hospital stays, and reduced discharge to acute rehab; Significantly increased risk of extension surgery
Chou et al., 2018 [31]	Retrospective Cohort	118	III	Patients with adult scoliosis	Minimally invasive VERSUS open fractional curve correction	MIS approach with significantly less instrumented and decompressed levels; similar clinical outcomes in both cohorts
Kasliwal et al., 2012 [32]	Retrospective Cohort	60	III	Patients with previous short-segment fusion for adult scoliosis VERSUS patients undergoing initial operation	Scoliosis deformity correction	No significant differences in complications, perioperative morbidity/mortality, and clinical outcomes
Zurbriggen et al., 1999 [33]	Case series	40	IV	Patients with degenerative lumbar scoliosis	Posterior instrumentation and fusion	Improvements seen in radiographic and clinical outcomes following surgical intervention
Feng et al., 2015 [34]	Prospective Cohort	159	III	Patients with isthmic spondylolisthesis	Posterolateral fusion VERSUS Posterior lumbar interbody fusion	PLIF better at augmenting lumbar lordosis and aiding with the restoration of spinopelvic parameters
Johnson et al., 2013 [35]	Retrospective Cohort	22	III	Patients with degenerative lumbar disc disease	Extreme lateral interbody fusion	Significant improvements in segmental lordosis, scoliotic Cobb angle, and clinical outcomes
Anand et al., 2008 [36]	Retrospective Cohort	12	IV	Patients with degenerative lumbar scoliosis	Circumferential MIS fusion of deformity	Post-operative improvements seen in coronal Cobb angle, VAS score, and TIS score.
Hasegawa and Homma 2003 [37]	Case Series	23	IV	Patients with degenerative lumbar kyphoscoliosis	Multi-level posterior lumbar interbody fusion	Significant improvements in JOA score, Cobb angle, and torsional deformity noted post-operatively
Dakwar et al., 2010 [38]	Retrospective Cohort	25	IV	Patients with adult degenerative deformity	Lateral interbody fusion via transpsoas approach	VAS and ODI improvements seen post-operatively
Lee et al., 2016 [39]	Prospective Cohort	32	III	Patients with adult degenerative deformity	Lateral and Anterior lumbar interbody fusion with posterior fixation	ALIF levels with greater post-op segmental lordosis compared to LLIF levels; also noted greater increase in segmental lordosis; sagittal parameters all improved post-operatively; see worse parameters at follow-up, but still improved compared to pre-op
Ahlquist et al., 2018 [40]	Retrospective Cohort	164	III	Patients undergoing lumbar fusion	Anterior VERSUS Lateral VERSUS transforaminal VERSUS posterior lumbar interbody fusion	ALIF and LLIF with significant improvements in segmental lordosis, anterior and posterior disc heights, and foraminal height; ALIF and LLIF outperformed PLIF in improvements seen post-op; ALIF only technique to significantly increase proportion of PI-LL < 10°
Anand et al., 2010 [41]	Retrospective Cohort	28	IV	Patients with adult scoliosis	Minimally invasive correction of deformity, 3+ levels	Improvements in VAS, TIS, ODI, and SF-36 scores; lower perioperative morbidity
Lo et al., 2015 [42]	Retrospective Cohort	973	III	Patients with adult degenerative deformity	Single-level fusion	Mini-Open and MIS with lower EBL, VAS, LOS, and infections; longer surgery time for both
Seng et al., 2013 [43]	Retrospective Cohort	80	III	Patients with adult degenerative deformity	Open VERSUS minimally invasive transforaminal lumbar interbody fusion	Perioperative variables—MIS had higher fluoroscopic time, less blood loss and morphine usage, and less time to ambulation and less LOS; all groups with significant improvements in patient-reported outcomes—no significant differences between groups; all groups with significant fusion by 5 years—open TLIF had nonsignificantly higher rates within 6 months and 2 years
Alimi et al., 2015 [44]	Retrospective Cohort	23	III	Patients with single-level unilateral vertical foraminal stenosis with radicular pain	Single-level extreme lateral interbody fusion	Significant increases in foraminal height and disc height; significant decrease in coronal Cobb, VAS-Leg, VAS-Buttock, and VAS-Back
Tani et al., 2022 [45]	Retrospective Cohort	36	III	Patients with adult spinal deformity	Anterior column reconstruction, lateral lumbar interbody fusion, and percutaneous pedicle screw fixation	Patients had significantly increased lumbar lordosis, thoracic kyphosis, and segmental disc angles after intervention; significantly decreased PI-LL and spino-vertebral angle; significant increases in disc heights, foraminal height, and cross-sectional area; decreases in ligamentum flavum thickness and disc bulge thickness; significant decrease in ODI
Elsamadicy et al., 2017 [46]	Retrospective cohort	874	III	Patients with adult spinal deformity	Spinal fusion alone VERSUS spinal fusion with laminectomy	Laminectomy cohort with increased blood loss, blood transfusions, and durotomies intra-op; higher rate of ICU post-op
Kanayama et al., 2007 [47]	Retrospective cohort	56	III	Patients with adult spinal deformity	Graf ligamentoplasty	No significant differences in segmental lordosis—see a reduction in range of motion at the operative level; significant improvement in JOA scores from pre-op to follow-up. Unfavorable outcomes in degenerative scoliosis and lateral listhesis
Di Silvestre et al., 2010 [48]	Retrospective cohort	29	III	Patients with adult spinal deformity	Dynamic Stabilization without fusion	Significant improvements in ODI, RDQ, and VAS Back and Leg; significant improvements in scoliosis, Cobb angle, lateral listhesis, and anterior vertebral translation
Zhao et al., 2020 [49]	Retrospective cohort	16	III	Patients with adult lumbar degenerative scoliosis	Short-segment decompression and fusion WITH proximal segment stabilization	Significant changes seen in radiographic measures as well as in VAS Back + Leg and ODI

## Data Availability

No new data were created or analyzed in this study. Data sharing is not applicable to this article.

## Supplementary Materials

The following are available online at https://www.mdpi.com/article/10.3390/jcm13041030/s1, Table S1: Risk of Bias Assessment.
