# Supplementary material for: Limited Intervention in Adult Scoliosis—A Systematic Review
Source: J Clin Med. 2024 Feb 11;13(4):1030. doi: 10.3390/jcm13041030 (PMC10888624; doi:10.3390/jcm13041030)
Supplement: Supplementary file 1 [file jcm-13-01030-s001.zip › jcm-2808512-supplementary.pdf]

Supplementary Table S1. Risk of Bias Assessment

Study	Bias due to confounding	Bias in selection of participants into the study	Bias in classification of interventions	Bias due to deviations from intended interventions	Bias due to missing data	Bias in measurement of outcomes	Bias in selection of the reported result	Overall Bias
Nakajima et al. 2022	Low	Low	Low	Low	Low	Low	Low	Low
Cho et al. 2008	Low	Low	Low	Low	Low	Low	Low	Low
Liu et al. 2009	Moderate	Low	Low	Low	Low	Low	Low	Moderate
Wang et al. 2016	Moderate	Low	Low	Low	Low	Low	Low	Moderate
Li et al. 2021	Moderate	Low	Low	Low	Low	Low	Low	Moderate
Song et al. 2022	Moderate	Low	Low	Low	Low	Low	Low	Moderate
Khalifé et al. 2023	Moderate	Low	Low	Moderate	Low	Low	Low	Moderate
Schairer et al. 2013	Moderate	Low	Low	Moderate	Low	Low	Low	Moderate
Hart et al. 2013	Moderate	Low	Low	Low	Low	Low	Low	Moderate
Hart et al. 2014	Serious	Low	Low	Moderate	Low	Low	Low	Serious
Isaacs et al. 2010	Moderate	Low	Low	Low	Low	Low	Low	Moderate
Pateder et al. 2008	Low	Low	Low	Low	Low	Low	Low	Low
Frazier et al. 1997	Moderate	Low	Low	Low	Low	Low	Low	Moderate
Minamide et al. 2017	Low	Low	Low	Low	Moderate	Low	Low	Moderate
Aoki et al. 2015	Low	Low	Low	Low	Low	Low	Low	Low
Bari et al. 2021	Moderate	Low	Low	Moderate	Moderate	Low	Low	Moderate
Lugue et al. 2020	Low	Low	Low	Low	Moderate	Low	Low	Moderate
Pugely et al. 2014	Moderate	Low	Low	Low	Low	Low	Low	Moderate
Kapetanakis et al. 2017	Low	Low	Low	Low	Low	Low	Low	Low
Kim et al. 2021	Serious	Low	Low	Low	Low	Low	Low	Serious
Bai et al. 2017	Serious	Low	Low	Low	Low	Low	Low	Serious
Telfeian et al. 2018	Low	Low	Low	Low	Low	Low	Low	Low
Madhavan et al. 2016	Low	Low	Low	Low	Low	Low	Moderate	Moderate
Brodke et al. 2013	Moderate	Low	Low	Low	Low	Low	Low	Moderate
Hasan et al. 2019	Moderate	Low	Low	Low	Low	Low	Low	Moderate

Uribe et al. 2017	Low	Low	Low	Low	Low	Low	Low	Low
Deukmedjian et al. 2013	Moderate	Low	Low	Low	Low	Low	Moderate	Moderate
Park et al. 2013	Moderate	Low	Low	Low	Low	Low	Low	Moderate
Liang et al. 2020	Moderate	Low	Low	Moderate	Low	Low	Low	Moderate
Amara et al. 2019	Moderate	Low	Low	Low	Low	Low	Low	Moderate
Chou et al. 2018	Low	Low	Low	Low	Low	Low	Low	Low
Kasliwal et al. 2012	Low	Low	Low	Low	Low	Low	Low	Low
Zurbriggen et al. 1999	Low	Low	Low	Low	Low	Low	Low	Low
Feng et al. 2015	Moderate	Low	Low	Low	Low	Low	Low	Moderate
Johnson et al. 2013	Moderate	Low	Low	Low	Moderate	Low	Low	Moderate
Anand et al. 2008	Low	Low	Low	Serious	Low	Low	Low	Serious
Hasegawa and Homma 2003	Low	Low	Low	Low	Low	Low	Low	Low
Dakwar et al. 2010	Serious	Low	Low	Moderate	Low	Low	Low	Serious
Lee et al. 2016	Moderate	Low	Low	Moderate	Low	Low	Low	Moderate
Ahlquist et al. 2018	Moderate	Low	Low	Moderate	Low	Low	Low	Moderate
Anand et al. 2010	Moderate	Low	Low	Low	Low	Low	Moderate	Moderate
Lo et al. 2015	Low	Low	Low	Low	Low	Low	Moderate	Moderate
Seng et al. 2013	Low	Low	Low	Low	Low	Low	Moderate	Moderate
Alimi et al. 2015	Moderate	Low	Low	Moderate	Low	Low	Low	Moderate
Tani et al. 2022	Moderate	Low	Low	Low	Low	Low	Low	Moderate
Elsamadicy et al. 2017	Moderate	Low	Low	Low	Low	Low	Low	Moderate
Kanayama et al. 2007	Moderate	Low	Low	Low	Low	Low	Moderate	Moderate
Di Silvestre et al. 2010	Moderate	Low	Low	Low	Low	Low	Low	Moderate
Zhao et al. 2020	Moderate	Low	Low	Low	Low	Low	Low	Moderate
